# Fiber Bragg Grating Array for Shape Reconstruction in Structural Elements

**DOI:** 10.3390/s22176545

**Published:** 2022-08-30

**Authors:** Edson A. Souza, Leandro C. Macedo, Anselmo Frizera, Carlos Marques, Arnaldo Leal-Junior

**Affiliations:** 1 Mechanical Engineering Department, Federal University of Espírito Santo, Vitória 29075-910, Espirito Santo, Brazil; 2 Graduate Program in Electrical Engineering, Federal University of Espírito Santo, Vitória 29075-910, Espirito Santo, Brazil; 3 Department of Physics and I3N, Campus Universitário de Santiago, University of Aveiro, 3810-193 Aveiro, Portugal

**Keywords:** fiber Bragg gratings, optical fiber sensors, shape reconstruction

## Abstract

This paper presents the development, analysis and application of a fiber Bragg grating (FBG) array for two-dimensional (2D) shape reconstruction in a cantilever beam. The structural elements made of Pinus wood and Nylon 6.0 were numerically analyzed using the finite element method for the strain distribution when constant loading is applied at the free end of the beam. In addition, the temperature compensation method is proposed to decouple the temperature cross-sensitivity in the deflection analysis. In this case, the temperature sensitivities of all sensing elements of the 5-FBG array were obtained. An additional FBG was encapsulated in a silicone mold for increased sensitivity and positioned in the clamping point in which deflection was negligible. Temperature compensation was achieved considering the temperature measured by the silicone-embedded FBG (sensitivity of 27.78 pm/°C) and the sensitivity of all five FBGs of the deflection-sensing array (9.14 pm/°C ± 0.33 pm/°C). In the deflection experiments, the sensors presented a high linearity, in which a determination coefficient (R^2^) higher than 0.995 was obtained in all of the analyzed cases. Furthermore, the 2D shape construction using the proposed sensor approach resulted in the elastic line estimation for all analyzed beams, where the experimental results were in agreement with the theoretical and numerical analysis with a R^2^ higher than 0.99 in all of the analyzed cases. Therefore, the proposed sensor array is a feasible approach for real-time shape reconstruction of structural elements with the advantages related to the possibility of direct embedment in the measured structure.

## 1. Introduction

A mechanical or civil structure is subjected to different types of stress throughout its lifespan. These stresses can range from static to dynamic (e.g., car traffic atop a bridge, wind gusts, and earthquakes). In order to ensure the proper operation of these structures, health monitoring is necessary, implying greater control and security. As a result of this monitoring, it is possible to assess real operational conditions and determine whether there are any structural issues that were not identified during the construction phase. By monitoring critical points caused by wear and tear and corrosion, more assertive preventive decisions can be made. The mechanical properties are compromised at these points, which require attention and aim to avoid structural issues [1].

To monitor the structural integrity of civil structures, such as bridges and tunnels, FBG sensors are adaptable, accurate, and low-cost. In terms of adaptability, these sensors can be integrated into the surface of an existing structure or embedded during its construction [1,2].

Due to their characteristics, optical sensors provide an alternative to conventional electromechanical sensors, complementing some of their limitations. Among its key characteristics are its small size and low weight, requiring little space for implementation, immunity to electromagnetic interference, immunity to corrosion, intrinsic safety, an inability to ignite in flammable environments, multiplexing, and high sensitivity [3,4].

Due to the photosensitivity of optical fibers, it is possible to manufacture FBG sensors, which entail the inscription of Bragg gratings along the longitudinal axis of the optical fiber core for selectively reflecting wavelength [5]. A Bragg grating reflects a small portion of the signal spectrum of the injected light beam, whose central wavelength is called the Bragg wavelength $\lambda_{B}$ [6]. Equation (1) defines the Bragg wavelength.

(1) $$\lambda_{B}=2n_{eff}\Lambda$$

In Equation (1), $n_{eff}$ is the effective refractive index of the optical fiber core and Λ is the periodicity generated in the recorded Bragg gratings.

In response to external physical parameters (such as deformation, temperature, and pressure), FBG sensors display variations in the Bragg wavelength. Typically, the measurand is an external physical parameter, whose information is encoded by the Bragg wavelength variation. Using proper sensor characterization and specific correlation equations, information can be decoded from a sensor [7,8].

In general, the shape reconstruction is performed from reconstruction algorithms, which is achieved from strain, deflection or angle measurements from distributed or quasi-distributed sensors [9]. In this case, it is possible to use the same algorithms for shape reconstruction using electronic strain gauges or any other non-distributed system for the optical fiber sensor-based shape reconstruction [10]. The advantages of using the optical fiber sensors in shape reconstruction applications are related to their compactness and their capability of embedment in different materials without significant variations to the material structure and physical behavior [11]. Moreover, the multiplexing capabilities of FBGs are a crucial feature for their use in shape reconstruction methods, since many sensors can be distributed in the structure using a single optical fiber cable [7]. Such advantages motivated the use of optical fiber sensors in shape reconstruction approaches, where 2D or single-plane reconstruction is achieved by using FBG sensors [12] as well as the radially positioned optical fibers for 3D reconstruction [13] or cladding mode-based [14] and tilted FBG structures [15]. It is also important to mention that optical reflectometry techniques can be applied for high-resolution shape reconstructions in biomedical applications [16].

In this paper, five distributed FBG sensors are integrated along a Pinus Wood beam and a Nylon 6.0 beam. Sensors estimate the elastic line that describes the deflection over the entire length of the beam by reproducing the tests with load applications. Additionally, we have used the temperature compensation method for cross-sensitivity and performed a computational analysis of the beams through numerical simulations.

## 2. Materials and Methods

Since Meltz and Hill demonstrated that it is possible to periodically modulate the refractive index of optical fiber cores externally, various techniques for external recording of gratings inside the core have emerged. These techniques are classified as either interferometric or non-interferometric [17]. Due to the small orders of dimensions involved in this technology, the engraving procedure requires a highly precise and stable system. The phase mask technique is used in this work and is one of the most commonly used techniques.

The phase mask consists of an optical diffraction element made of silica and is characterized by periodic depressions, $\Lambda_{pm}$, along its longitudinal axis. Using procedures, it is manufactured with the aim of minimizing the order *m* = 0 [18]. Then, there is an interference pattern in the Bragg gratings region of the optical fiber core when the UV beam falls perpendicularly on the phase mask. The beams are diffracted in the order of m = −1 and m = 1, which results in a spatial superposition of the beams. Since the spatial coherence of the laser must be greater than the distance between the phase mask and fiber core, the optical fiber (without the coating that involves the shell and core) must be positioned right after the phase mask. In this manner, the coating is prevented from adhering to the phase mask [19,20].

### 2.1. Beam Materials Analysis

Two beams were used in the shape reconstruction experiment, one beam made of Pinus Wood and a second beam made of Nylon 6.0. Such materials were chosen due to their differences in their material features, which result in a wider evaluation of the proposed optical fiber sensing array at different material conditions. For this reason, not only were different materials chosen, but also different transverse areas and moments of inertia. The beams were positioned in the single cantilever configuration with the load applied to the beam’s end. The physical and dimensional properties of the beams used are presented in Table 1.

Here, *E* represents the modulus of elasticity of the beam materials, according to the ISO 527 standard determining the property for Nylon 6.0 and, according to the ABNT MB26/53 (NBR6230/85) standard to determine this property for Pinus Wood. *I* represents the moment of inertia and *L* represents the length of the beams. It is important to mention that the cross section of the nylon beam has a circular shape, with a diameter of 16 mm, whereas the wooden beam has a rectangular cross section, with 101 mm × 11 mm. The cross-section dimensions, as well as the thickness of the wooden beam, leads to the necessity of eccentricity minimization on the load application to avoid the effects of torsion on the sensors.

### 2.2. Temperature Cross-Sensitivity Analysis

In cases of structural integrity analysis with a significant temperature variation, a temperature cross-sensitivity occurs, where two parameters (temperature and strain) influence the Bragg wavelength variation. In cases of structural analysis, we have both the temperature influencing the wavelength variation and the deformation experienced by the FBG sensor [21]. In this case, the following equation indicates the Bragg wavelength variation as a function of strain and temperature:

(2) $$\Delta \lambda_{B}=2\left(\Lambda \frac{\partial n_{eff}}{\partial \varepsilon }+n_{eff}\frac{\partial \Lambda }{\partial \varepsilon }\right)\Delta \varepsilon +2\left(\Lambda \frac{\partial n_{eff}}{\partial T}+n_{eff}\frac{\partial \Lambda }{\partial T}\right)\Delta T$$

where Δε and Δ*T* are the variations related to strain and temperature, respectively. However, in the case of the current work, we will analyze the deflection experienced by the sensor and the temperature, defined by the equation below for simplicity.

(3) $$\Delta \lambda_{B}=S_{v}\Delta v+S_{T}\Delta T$$

*S_v_* and *S_T_* are the sensitivity of the sensors to deflection and temperature respectively, being defined in the characterization step of the FBGs. The term Δ*v* represents the variation to deflection, measured by the sensor.

**Figure 1:**
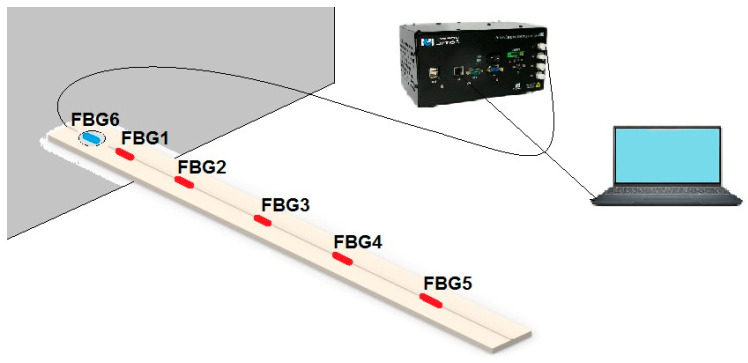
Experimental setup of the beam characterization method.

It is important to mention that the temperature variation also results in the thermal expansion of the beams. Therefore, for applications in which there are large temperature variations, it would be necessary to also consider the thermal distribution along the beam. In these cases, another sensor array for temperature assessment (isolated from strain variations) would be necessary to evaluate the temperature distribution in the beam. However, in the experiments presented in this paper, the temperature variation is below 1 °C (as shown in Section 3). Such small temperature variation does not lead to significant thermal expansion of the Pinus Wood and Nylon 6.0 materials, especially when considering the transverse area of these structures. For this reason, the temperature compensation is achieved by using only one FBG for temperature assessment (FBG 6) positioned on the beam as shown in Figure 1.

### 2.3. FBGs Fabrication

The production of the FBGs was accomplished with a pulsed Q-switched Nd:YAG laser system (LOTIS TII LS-2137U laser), emitting at 266 nm with a pulse-repetition rate of 10 Hz and 27 J of pump energy. The inscription setup is based on the phase mask technique, in which the laser beam goes through four mirrors and a plano-convex lens before reaching the phase mask [22]. The laser beam, with a pulse duration of 8 ns, has a circular profile with approximately 8 mm in diameter and a maximum divergence of 1 mrad. The phase masks employed in the grating inscription allow the production of FBGs up to 10 mm in length and with different pitches for the C-band.

A reference FBG was placed in a region without strain application to detect only temperature variations during the tests as a tool for the temperature compensation in case of cross sensitivity.

### 2.4. Numerical Analysis

For the numerical analysis of the beam, the finite element method was used, since it is a powerful tool in the numerical solution of systems with complex geometries. The method consists of discretizing a continuum system of complex geometry into several elements of simple geometries, making the solution relatively simple and close to the continuum. The discretization and optimization of the mesh create a solution closer to a continuous medium [23]. The finite element method was applied to the nylon and the wooden beam, creating virtual models of the beams and simulating the condition in which they were submitted in the tests, using the Ansys^®^ Workbench software. The configuration reproduced in the simulation was a cantilever beam with a load applied to the free end.

### 2.5. Experimental Setup

The FBG sensors were integrated directly onto the upper surface of both beams along the length and connected to the sm125 interrogator (Micron Optics, Atlanta, GA, USA) for data acquisition with a wavelength range from 1510 nm to 1590 nm, a wavelength accuracy of 10 pm, and an acquisition frequency of 2 Hz. The FBG sensor integration on the beams were performed by means of gluing them with a cyanoacrylate adhesive. In an additional channel of the interrogator, a reference FBG was connected to perform the temperature measurement. The interrogator connected to the laptop performed the data acquisition in this way. The experimental setup is presented in Figure 1.

The Figure 1 illustrates the Pinus Wood beam, and the experimental setup for the Nylon 6.0 beam was the same, but the wooden beam was replaced with the nylon beam.

In the temperature-response characterization, a controlled temperature oven (Ethik Technology) was used, where the FBGs were positioned inside. The temperature varied in the setpoint in a range of 25 °C to 45 °C with steps of 5 °C. It is also worth mentioning that the reference FBG was embedded in a silicone mold to achieve higher temperature sensitivity due to the larger thermal expansion coefficient of the silicone.

The FBG sensors were positioned with respect to the crimping point of the beams, which was considered to be the reference frame and x was the variable directed along the length. The positions of the FBGs sensors as a function of the beams’ length are shown in Table 2 along with their respective initial Bragg wavelengths.

The load application was done by adding elements with known masses at the end of the beam. The range of added masses was from 0.1 to 0.5 kg with a step increase of 0.1 kg for both beams. The Bragg wavelength variations due to the load increment were associated with the theoretical deflections (calculated by the superposition method) caused by the same magnitudes of the added loads. The deflection test described was reproduced three times to evaluate the sensors’ repeatability. It is worth mentioning that the choice of the three tests was due to the high repeatability of the FBG sensors, which resulted in only small deviations (below tens of picometers) between the experiments. Such behavior could also be verified in the experimental results, where the mean and standard deviations are shown, but the small standard deviation of the sensors was too small to be visible in the graph scale. Furthermore, different loading application positions were considered for the Pinus Wood and the Nylon 6.0. For the Pinus Wood, the loading application position was at 105.0 cm, which, compared with the position of FBG 5 (presented in Table 2), was between FBG 4 and FBG 5. On the other hand, the loading application position in the Nylon 6.0 beam was around 88.0 cm, i.e., between FBG 5 and the end facet of the beam’s free end. 

The shape reconstruction was obtained from the deflection data of each FBG in the array, since the FBG positioning on the beam was known, it was possible to evaluate the strain/deflection distribution along the structural element. Then, the shape reconstruction in a single plane was achieved by means of considering the deflection of the five measurement points (as presented in Section 3). Thus, considering this shape reconstruction method, the accuracy in the shape reconstruction will increase as the number of sensors increases. In addition, for a single-plane reconstruction, the sensors need to be positioned in the same longitudinal axis, which can be easily accomplished using all strain sensors in a single optical fiber cable. Otherwise, there would be errors in the single plane shape reconstruction due to misalignment between the sensors.

## 3. Results and Discussion

**Figure 2:**
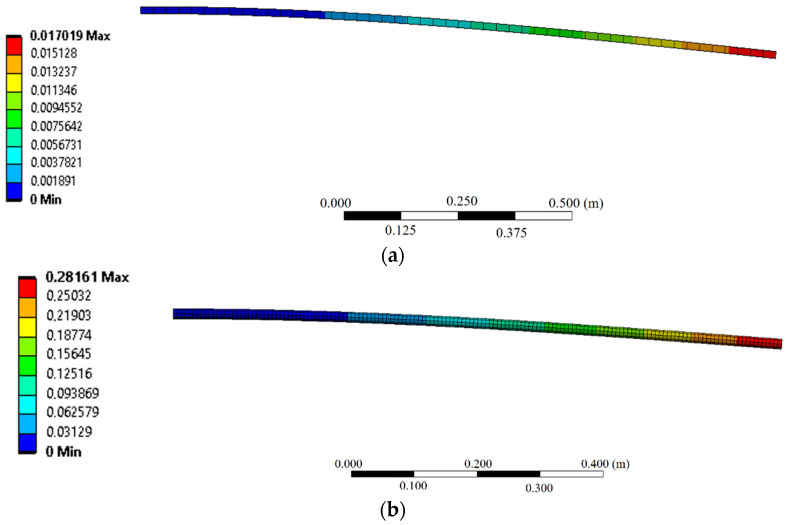
Numerical results of the displacement in the beams with the cantilever configuration for a 5 N load applied on the free end. (**a**) Wooden beam; and (**b**) Nylon 6.0 beam.

Figure 2a,b presents the results obtained in the numerical simulations in the wooden and nylon beams, respectively, for a load of around 5 N applied in the free end of the beam in the cantilever condition.

**Figure 3:**
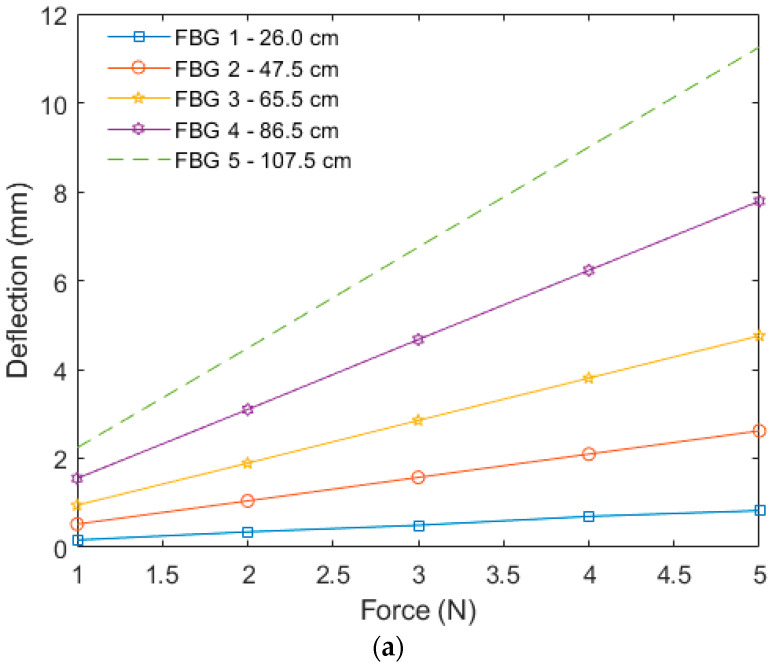

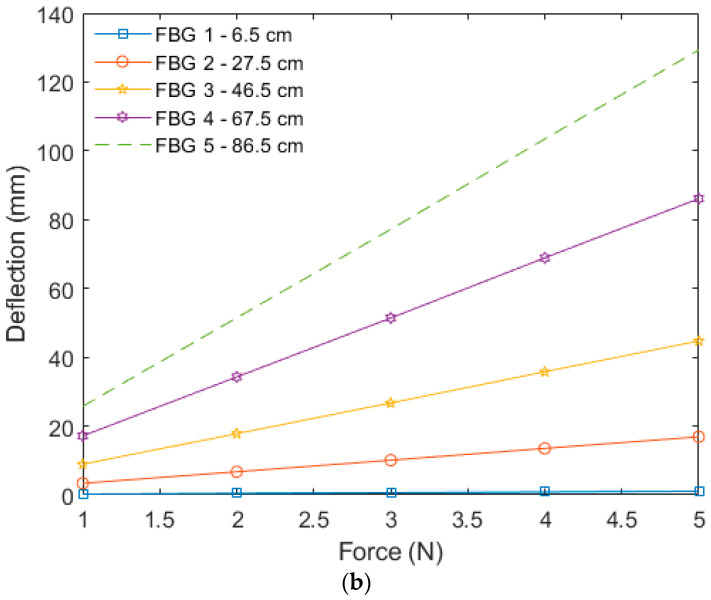
Numerical results of the displacement as a function of the applied force in the free end of the (**a**) Wooden beam; and (**b**) Nylon 6.0 beam.

The numerically calculated deflections at the free end of the beam for the loads of 1 N, 2 N, 3N, 4 N, and 5 N are presented in Figure 3 for Pinus Wood and Nylon 6.0, in Figure 3a and Figure 3b, respectively. The deflections presented in Figure 3 were obtained at different positions, where these positions were chosen as the positions of the FBG positioning in the experimental 2D shape reconstruction of the beam. The numerical results indicate that higher deflections were obtained closer to the free end of the beam, which was expected, since it was closer to the load application point. Furthermore, there was a linear relationship between the deflection and the load in all of the analyzed positions. Comparing the Pinus Wood and Nylon 6.0 beams, the nylon beam presented higher deflections, which was related to its material properties and cross-sectional geometry. 

**Figure 4:**
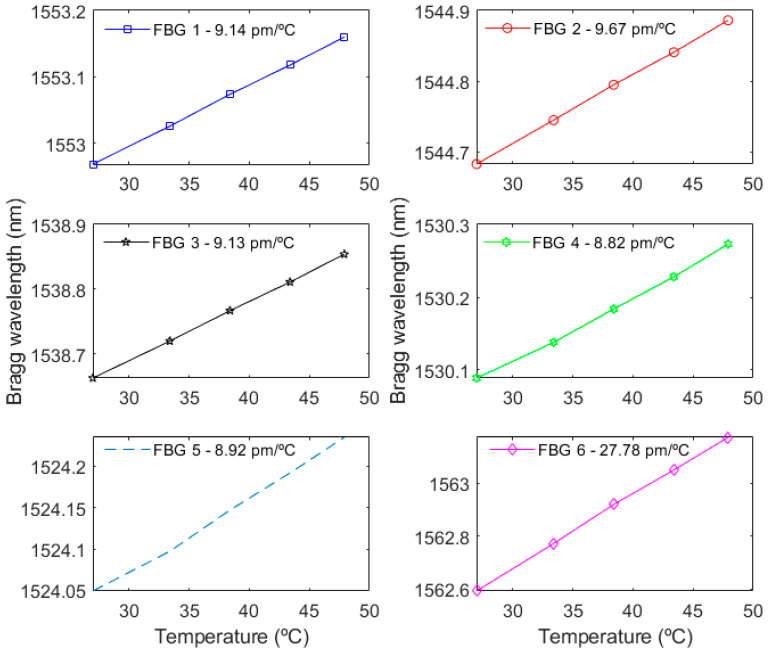
Bragg wavelength as a function of the temperature for each FBG.

After the numerical evaluations of the beam, the FBG array can be positioned in different positions along the beams to evaluate the strain distribution and deflections, which is used on the shape reconstruction of the structural element. However, the temperature variations can lead to errors in the strain distribution and shape reconstruction. Thus, the temperature response of the FBGs was evaluated prior to their application in the beams. Figure 4 presents the temperature characterization of the FBGs, where the five FBGs for the shape reconstruction of the beams presented similar temperature sensitivities 9.14 ± 0.33 pm/°C. 

It is worth mentioning that the FBG used as the temperature reference (the so-called FBG 6) had a higher temperature sensitivity (27.78 pm/°C) due to the silicone resin applied on the sensor embedment. This FBG was positioned on the region at which the beam was anchored, and such region was not subjected to strain and deflections. For this reason, FBG 6 was subjected only to the temperature variations and its higher temperature sensitivity enabled a temperature assessment with higher resolution, which was important for the temperature compensation in FBGs 1–5. In this case, the maximum temperature variation estimated by FBG 6 during the deflection tests was calculated as shown in Equation (4), where the maximum wavelength variation ${\left(\Delta \lambda_{b}^{max}\right)}_{T}$ was 3 pm. Dividing the maximum wavelength variation by the temperature sensitivity *S_T_* of the reference FBG, the maximum temperature variation Δ*T^max^* during the test was calculated as 0.108 °C.

(4) $$\Delta T^{max}={\left(\Delta \lambda_{b}^{max}\right)}_{T}/S_{T}=0.108{\;}^{\circ }C$$

The maximum temperature variation measured during both set of tests were obtained and were below 0.108 °C. We used the same methodology to measure the maximum temperature variation that occurred in the test with the wooden beam. The temperature variations measured by FBG 6 were applied in Equation (5) for the deflection estimation using FBGs 1–5, where the index *x* in Equation (5) represents the number of the FBG (1 to 5).

(5) $$\Delta v_{x}=\frac{\Delta \lambda_{B}-S_{T,x}\Delta T}{S_{v,x}}$$

**Figure 5:**
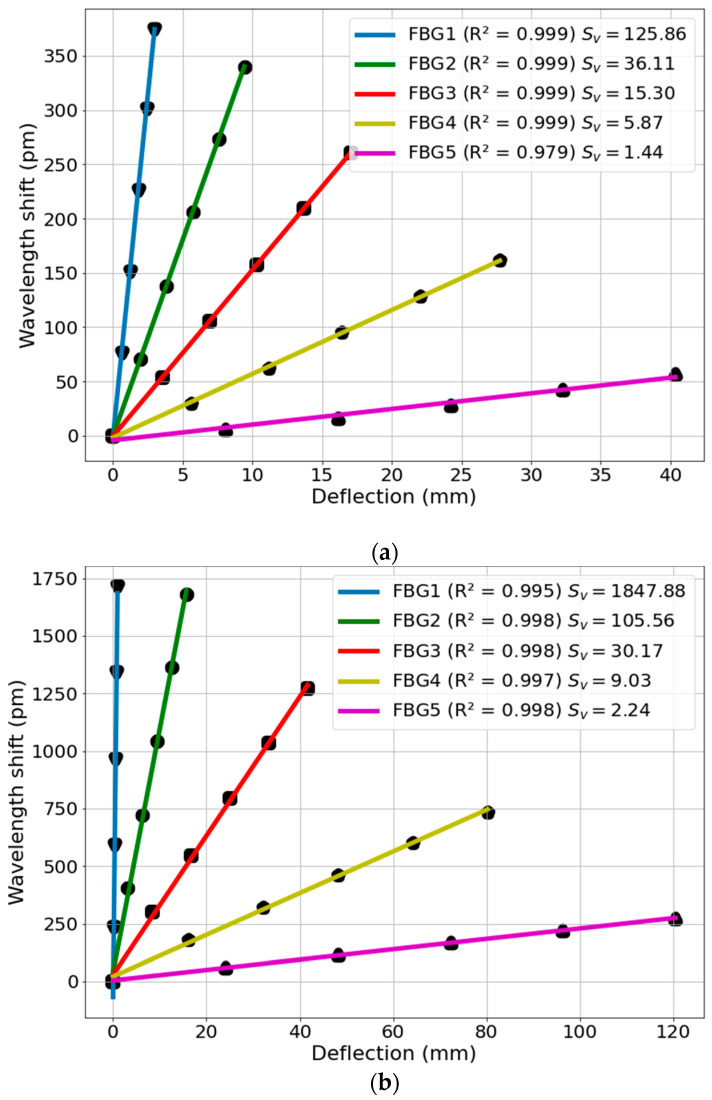
Experimental results of the wavelength shift for each FBG as a function of the deflection caused by the load applied at the free end of the (**a**) Wooden beam; and (**b**) Nylon 6.0 beam.

From the three tests reproduced for each beam, the average of the three variations of the Bragg wavelengths related to the application of each load was calculated. This procedure was performed for all FBGs subjected to deflection. The variations in Bragg wavelengths were associated at points with the deflections v(x) caused by the loads. Using the linear regression method, it was possible to generate the characterization curves of the FBGs, obtaining the sensitivity of the sensors to the deflection (*S_v_*) and the determination coefficient (R^2^). Figure 5a,b presents the results obtained from the characterizations for the Pinus Wood beam and for the Nylon 6.0 beam, respectively.

The R^2^ values in Figure 5 indicate a high linearity of the sensors as a function of deflection, with the adjustments closer to the ideal obtained by the FBGs integrated into the wooden beam, except for the characterization of FBG 5, which presented an R^2^ equal to 0.979. It is worth noting that the sensitivities decreased according to the positional order of the FBGs, with the highest sensitivity in FBG 1 and the lowest sensitivity in FBG 5. This can be explained by the fact that the localized deformations of the beams provoked a greater variation of wavelength in the regions closer to the anchored point.

After the temperature and deflection characterizations of the sensors, it was possible to measure the deflection obtained by each FBG in different load situations for both beams, for the elastic line assessment and 2D shape reconstruction of the different structural elements.

**Figure 6:**
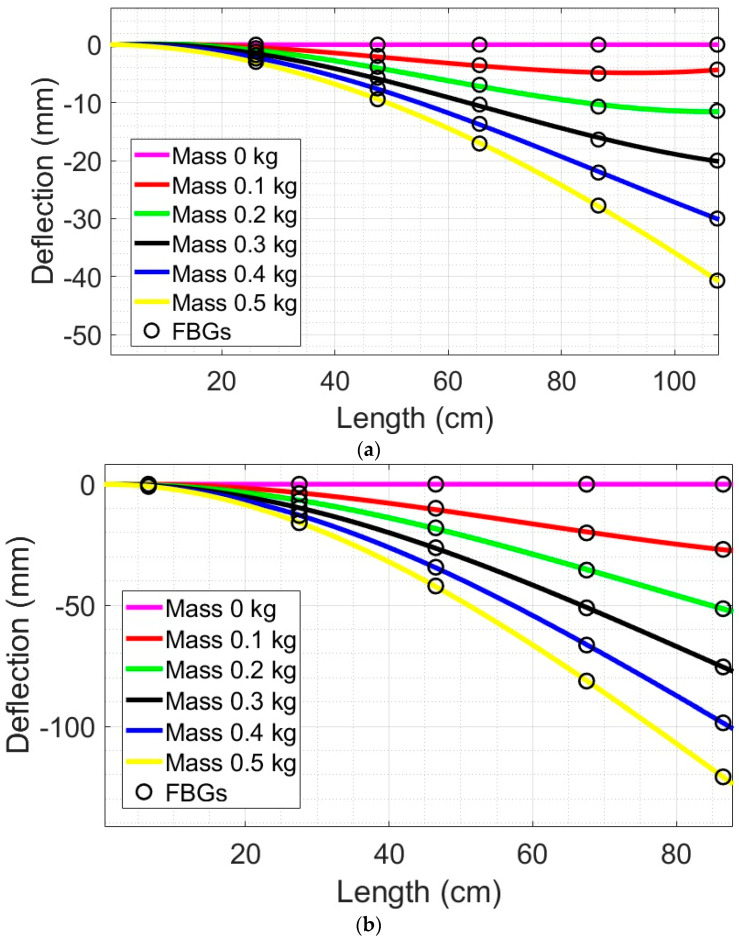
Elastic lines and 2D shape reconstruction at different load conditions for (**a**) Wooden beam; and (**b**) Nylon 6.0 beam.

The elastic line that describes the deflection over the entire length of the beam was estimated through the points measured by the sensors. The deflection variation over the length can be estimated from a polynomial equation of third degree, which is in accordance with the theoretical developments (as shown in Equation (6)), where the theoretical deflection along the beam length is estimated from the third-degree polynomial equation. The results of the estimated elastic lines for the wooden beam and for the nylon beam are shown in Figure 6a and Figure 6b, respectively.

As shown in Figure 6a,b, the elastic lines were estimated from the deflections measured by the sensors. The R^2^ values obtained in all regressions of Figure 6 were higher than 0.99, which indicated a high correlation between the experimental results and the numerical and analytical results. Thus, the proposed sensor system is capable of a 2D reconstruction of the beam regardless of the beam’s material, since both nylon and wooden beams were correctly reconstructed. However, it is possible to observe in Figure 6a that the Pinus Wood presented abnormal behavior in the longer lengths when subjected to smaller forces (around 1 N and 2 N). This can be related to the position of the load application, which is different to the one of the Nylon beam. In the case of the Pinus Wood, the load application occurred around 5 cm from the beam end, which could result in similar deflections between FBGs 4 and 5, especially for smaller forces.

The beam deflections measured by the FBGs sensors showed consistent results in a comparative analysis between the beams, i.e., similar behavior was obtained in all analyzed beams. The deflection was inversely proportional to the elastic modulus, i.e., higher elastic modulus led to smaller deflections (under the same load and geometry conditions) and vice-versa. The Nylon 6.0 beam had an elastic modulus that was lower than the one of Pinus Wood, which resulted in the higher deflections on the Nylon 6.0 beam, as shown in Figure 6b, when compared with the ones of the Pinus Wood presented in Figure 6a. In addition, the position of the FBG along the beam length was directly proportional to the deflection of the beam. Such a feature was verified in the experiments with the higher deflections measured by the sensors with longer distances to the crimping point of the beam.

## 4. Conclusions

This paper presented an FBG array for the 2D shape reconstruction in cantilever beams of different materials. The FBG sensors were positioned along the beam at different positions, but in the same plane. Theoretical and numerical analyses were performed to estimate the deflection of the different beams made of Nylon 6.0 and Pinus Wood. A temperature compensation was performed considering the temperature sensitivity of all sensors. The experimental results indicate the feasibility of the method in which a high correlation (higher than 0.99) was obtained for all of the analyzed cases. In addition, the difference in the value of the modulus of elasticity between the beams had more impact on deflection than the length. The deflection sensitivities of the sensors decreased according to the positional order of the FBGs, with the highest sensitivity of FBG1 and the lowest sensitivity of FBG5. This can be explained by the fact that the localized deformations of the beams cause a greater wavelength variation in the regions closer to the crimping, which together with the deflection values for the characterization of the FBGs, offers the characteristic observed in the characterization results of the sensors. FBG5 integrated to the wooden beam presented the lowest R^2^ value obtained by the FBGs. However, the behavior of the elastic lines of the other cases converged approximately to the same shape of the analyzed solutions described. The numerical and theoretical simulation results converged at all points of analysis with the experimental results obtained with the proposed FBG sensors. Therefore, the proposed sensor approach can be readily applied on the structural monitoring and shape reconstruction of different industrial elements, civil structures, geotechnical approaches, aircrafts, robotics, and even in biomechanics. Future works include the 3D shape reconstruction of the beams by using sensors radially positioned in the beam, the analysis at different configurations, and the analysis of strain distribution and resonances under harmonic loads.

## Figures and Tables

**Table 1:** Physical and dimensional properties of the beams.

Properties	Pinus Wood	Nylon 6.0
Elasticity Modulus (*E*)	6463.0 MPa	3200 MPa
Moment of Inertia (*I*)	1.12 × 10^−8^ m^4^	3.22 × 10^−9^ m^4^
Beam length (*L*)	139 cm	96.5 cm

**Table 2:** FBGs initial wavelength and positions on the wooden and nylon beams.

Properties	FBG 1	FBG 2	FBG 3	FBG 4	FBG 5
Pinus Wood—Position	26.0 cm	47.5 cm	65.5 cm	86.5 cm	107.5 cm
Nylon 6.0—Position	6.5 cm	27.5 cm	46.5 cm	67.5 cm	86.5 cm
Initial Bragg wavelength	1552.9 nm	1545.7 nm	1539.4 nm	1530.4 nm	1524.1 nm

## Data Availability

Data available upon reasonable request.
